# Oridonin suppresses gastric cancer SGC‐7901 cell proliferation by targeting the TNF‐alpha/androgen receptor/TGF‐beta signalling pathway axis

**DOI:** 10.1111/jcmm.17841

**Published:** 2023-07-11

**Authors:** Shiyong Gao, Huixin Tan, Dan Li

**Affiliations:** ^1^ Drug Engineering and Technology Research Center Harbin University of Commerce Harbin China; ^2^ Department of Pharmacy Fourth Affiliated Hospital of Harbin Medicine University Harbin China; ^3^ Heilongjiang Provincial Key Laboratory of Tumor Prevention and Antitumor Drugs Harbin China

**Keywords:** AR signalling pathway, gastric cancer, oridonin, TGF‐β signalling pathway, TNF‐α signalling pathway

## Abstract

Statistics provided by GLOBOCAN list gastric cancer as the sixth most common, with a mortality ranking of third highest for the year 2020. In China, a herb called *Rabdosia rubescens* (Hemsl.) H.Hara, has been used by local residents for the treatment of digestive tract cancer for hundreds of years. Oridonin, the main ingredient of the herb, has a curative effect for gastric cancer, but the mechanism has not been previously clarified. This study mainly aimed to investigate the role of TNF‐alpha/Androgen receptor/TGF‐beta signalling pathway axis in mediating the proliferation inhibition of oridonin on gastric cancer SGC‐7901 cells. MTT assay, cell morphology observation assay and fluorescence assay were adopted to study the efficacy of oridonin on cell proliferation. The network pharmacology was used to predict the pathway axis regulated by oridonin. Western blot assay was adopted to verify the TNF‐α/Androgen receptor/TGF‐β signalling pathway axis regulation on gastric cancer by oridonin. The results showed Oridonin could inhibit the proliferation of gastric cancer cells, change cell morphology and cause cell nuclear fragmentation. A total of 11signaling pathways were annotated by the network pharmacology, among them, Tumour necrosis factor alpha (TNF‐α) signalling pathway, androgen receptor (AR) signalling pathway and transforming growth factor (TGF‐β) signalling pathway account for the largest proportion. Oridonin can regulate the protein expression of the three signalling pathways, which is consistent with the results predicted by network pharmacology. These findings indicated that oridonin can inhibit the proliferation of gastric cancer SGC‐7901 cells by regulating the TNF‐α /AR /TGF‐β signalling pathway axis.

## 1 INTRODUCTION

*Rabdosia rubescens* (Hemsl.) H.Hara, a small shrubs of the genus Camellia in the labiaceae, is naturally distributed in the Yellow River and Yangtze River basins of China. Local residents have been treating cancer with *Rabdosia rubescens* (Hemsl.) H.Hara for decades in the Taihang and Wangwu mountain areas, located in the Henan Province in the People's republic of China. In 1972, Chinese researchers discovered that *Rabdosia rubescens* (Hemsl.) H.Hara has a unique anti‐cancer effect on cardiac cancer, liver cancer and oesophageal cancer. In the 1980s, further research and development of *Rabdosia rubescens* (Hemsl.) H.Hara was carried out and the main anti‐cancer components of *Rabdosia rubescens* (Hemsl.) H.Hara were identified as oridonin(purity >99.5%, buy from National Institutes for Food and Drug Control). The 2000 and the more recent 2020 edition of ‘Pharmacopoeia of the People's Republic of China’ officially included *Rabdosia rubescens* (Hemsl.) H.Hara. *Rabdosia rubescens* (Hemsl.) H.Hara has been shown to remove heat and help detoxify, promote blood circulation, relieve pain and has also been used for the treatment of sore throats and lumps. Meanwhile, the 2020 edition of pharmacopoeia has also included *Rabdosia rubescens* (Hemsl.) H.Hara based tablets.^1^

Oridonin (Figure 2A), a 7,20‐Epoxy‐ent‐Kauren Diterpenoid, is an effective ingredient with anti‐cancer activity.^2^, ^3^ The chemical formula is C_20_H_28_O_6_, the molecular weight is 364.44, the melting point is between 248 and 250°C, the compound is colourless or light yellow and displays a physical structure similar to needle‐like crystals. The compound tastes bitter, is slightly soluble in water and completely soluble in methanol, ethanol, dimethyl sulfoxide and other organic solvents. The enkeone system, the D‐ring core pharmacophore, is closely related to the biological activity of ordonin and its anticancer activity may be lost if the enkeone system is removed.^4^ In order to improve the bioavailability of oridonin, a variety of preparations such as liposomes^5^, ^6^ microspheres^7^ and nanoparticles^8^, ^9^ have been developed.

Oridonin has previously been investigated for its therapeutic effects on the following types of caners: colon,^2^, ^3^, ^6^, ^10^, ^11^ breast,^12^, ^13^ pancreatic,^14^, ^15^ lung,^16^, ^17^, ^18^ gallbladder,^19^ prostate,^20^ ovarian,^21^ oesophageal,^22^ oral,^23^, ^24^ bladder,^25^ liver^26^ and other cancers. It has been reported that the inhibitory effect of oridonin on proliferation of gastric cancer is mainly related to inducing apoptosis and promoting autophagy,^27^, ^28^, ^29^ without further in‐depth discussions. Our previous study found that oridonin can block the G_2_/M phase of gastric cancer cells^30^ and induce apoptosis^31^ by affecting the expression of cyclinD1/CDK4 and cyclinA/CDK2 proteins.

**FIGURE 1:**
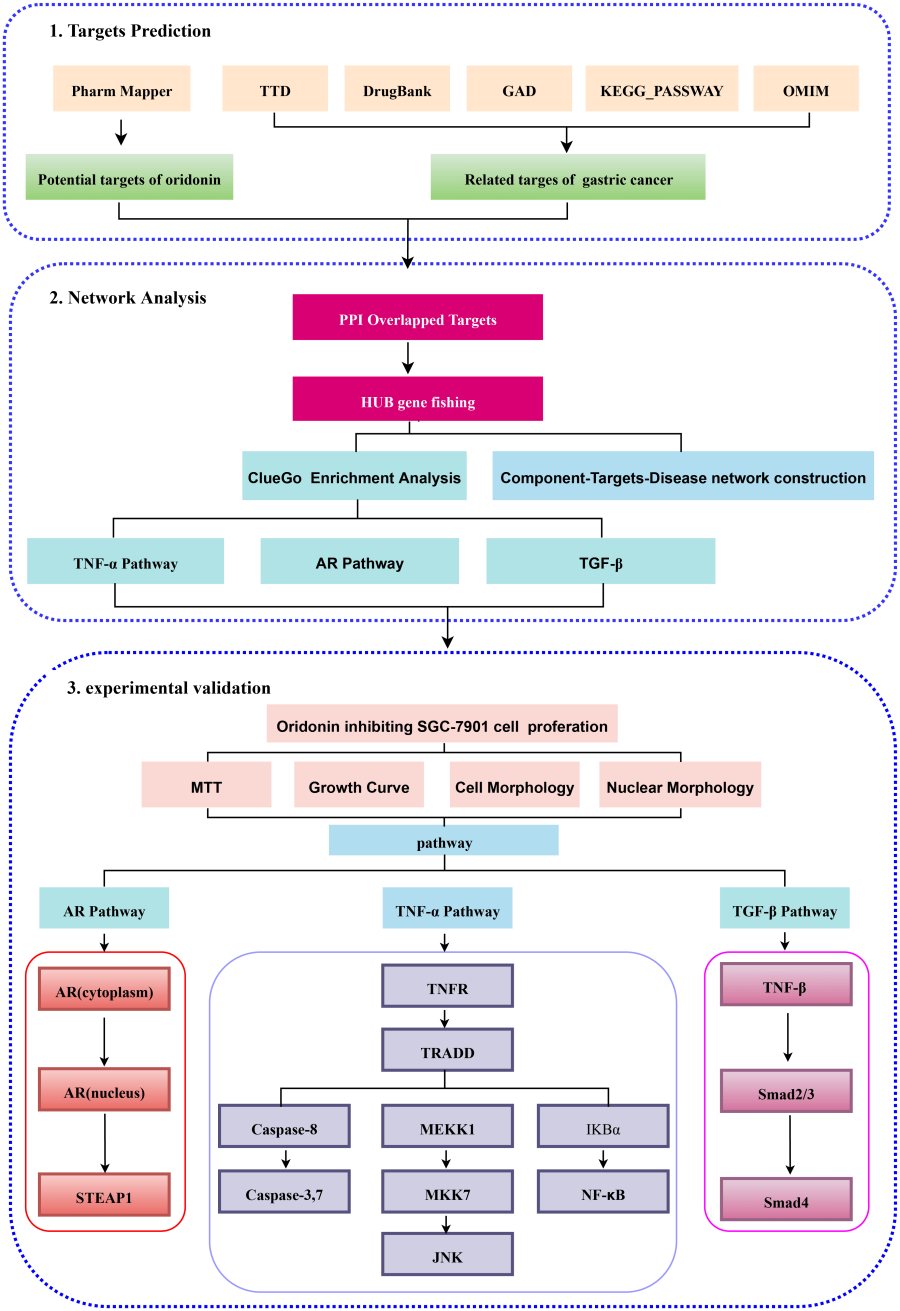
The schematic diagram of the research methodology and workflow of the present study of oridonin acting on gastric cancer.

Network pharmacology is a theory developed by Hopkins,^32^, ^33^ a British pharmacologist, in 2007 and is considered a new interdisciplinary subject. Based on systems biology and multidirectional pharmacology, network pharmacology gives a new idea for drug research from the perspective of a multi‐target strategy.^11^ Through the analysis and integration of big data, potential drug targets and signalling pathways can be predicted more comprehensively. In this study, we first confirmed that oridonin could inhibit the proliferation of gastric cancer cells. Then, the network pharmacology method was adopted to predict the signal pathway of oridonin for gastric cancer. Finally, experimental studies were carried out to study the predicted pathways and comprehensively reveal the mechanism of action of oridonin on gastric cancer. The research flow chart of this paper is shown in Figure 1.

## 2 MATERIALS AND METHODS

### 2.1 Cell line

Human gastric cancer SGC‐7901 cells were obtained from Heilongjiang Provincial Key Laboratory of Tumour Prevention and Antitumor Drug Research.

### 2.2 Chemicals and reagents

Oridonin (>98%) was purchased from National Institutes for Food and Drug Control of China. Adriamycin was purchased from Pharmacia Italia S.p.A., Gaggiano, Italy. Dimethyl sulfoxide (DMSO), trypsin, Tris, glycine, acrylamide, methylene diacrylamide were purchased from Sigma‐Aldrich. RPMI‐1640 cell culture medium was purchased from Gibco. Fetal bovine serum (FBS) was obtained from Sijiqing Hangzhou Bio Engineering Co., Ltd. Antibodies for caspase‐8, caspase‐3, caspase‐7, MEKK1, MEK7, JNK, IKBα, NF‐κB, AR, TGF‐β, Smad 2/3, TNF‐α, STEAP, Smad‐4, β‐actin and the secondary antibodies were purchased from Wanleibio.

### 2.3 In vitro cell viability assay

SGC‐7901 cells at the logarithmic growth stage were added into a 96‐well plate with 3.0 × 10^3^ cells/well. After incubation in the carbon dioxide incubator with 5% CO_2_ for 24 h, oridonin were added to cell suspensions and then incubated for another 72 h. This was followed by adding 0.5 mg/mL MTT solution to the suspensions, then incubated for 4 h in the carbon dioxide incubator. After this time, the liquid was poured out and 150 μL of DMSO was added to the suspensions, after which the optical density (OD) was detected by a microplate reader. The wavelength used for measurement was 570 nm, the IC_50_ value and inhibition rate were calculated.^31^, ^34^

### 2.4 Cell morphological and nuclei morphologicalchanges

Cell morphology was observed by inverted microscope after treating cells with oridonin. Morphological changes of the cell nuclei were examined by fluorescence analysis with a Hoechst 33258 stain solution. SGC‐7901 cells treated with oridonin for 24 h were fixed in 4% paraformaldehyde. Then, the cells were exposed to the Hoechst33258 stain solution for 30 min. Finally, the cells were washed three times in cold PBS, and the morphology of the cell nuclei was observed using a fluorescence microscope.

### 2.5 Analysis of oridonin on gastric cancer by network pharmacology

#### 2.5.1 Potential targets of oridonin

**FIGURE 2:**
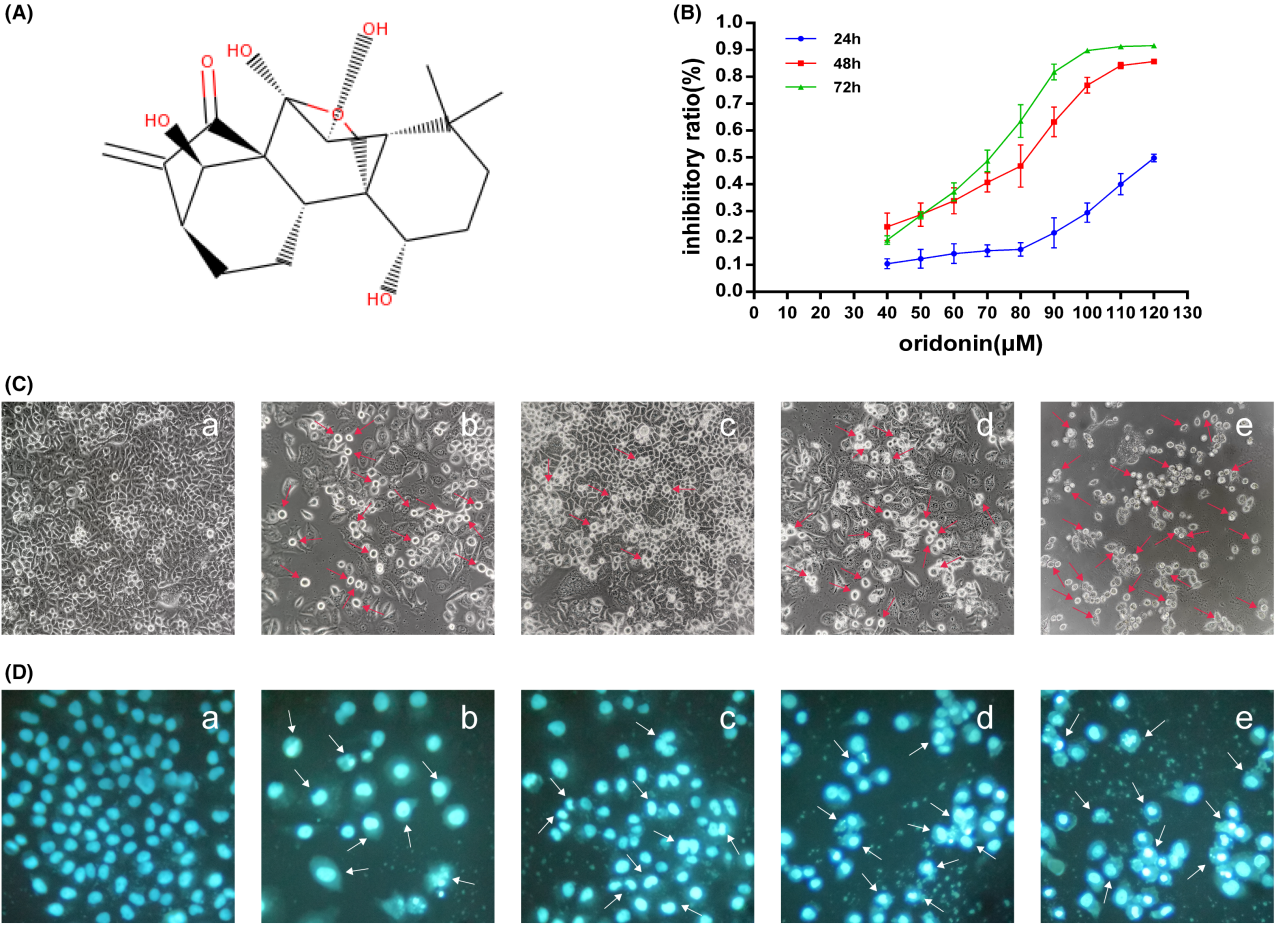
Effect of oridonin on proliferation of SGC‐7901 cells. (A) 2D chemical structural formula of oridonin. (B) The growth curve. (C) Cell morphology. a. Control; b. 0.35 μM ADR; c 30 μM oridonin; d 60 μM oridonin; e 90 μM oridonin. (D) Cell nuclear morphology. a. Control; b. 0.35 μM ADR; c. 30 μM oridonin; d. 60 μM oridonin; e. 90 μM oridonin.

**FIGURE 3:**
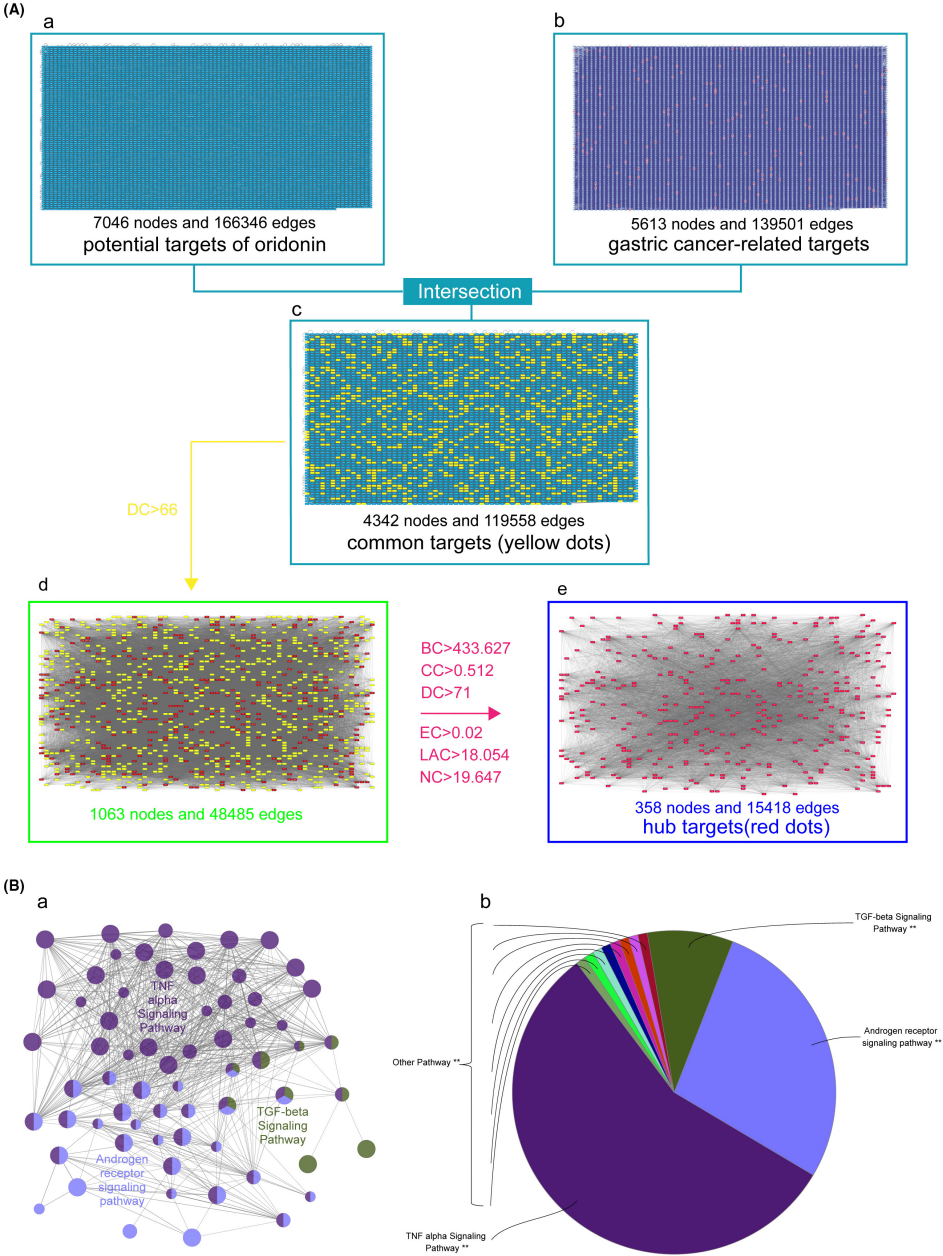
The fishing of Hub targets and the enrichment analysis of oridonin on gastric cancer. (A) The fishing of Hub targets. a. 7046 targets‐related of oridonin with 166346 edges; b 5613 target‐related of gastric cancer with 139501 edges; c 432 common targets of oridonin on gastric cancer with 119558 edges; d 1063 common targets (DC > 66) with 48485 edges; e 385 Hub targets with 15418 edges. (B) the pathway enrichment analysis of the Hub targets. a. Dot plot. b. Pie plot.

The 2D (Figure 2A) and 3D structures of oridonin were obtained from PubChem (https://pubchem.ncbi.nlm.nih.gov/). The mol2 file of oridonin was obtained from ZINC (https://zinc.docking.org). The structural information was used to look for potential targets of oridonin from PharmMapper (http://lilab.ecust.edu.cn/, updated May 29, 2018). Uniport (http://www.uniprot.org/) was adopted to standardize the nomenclature.

#### 2.5.2 Related targets of gastric cancer

The gastric cancer‐related targets were obtained from five databases: Drugbank (https://www.drugbank.ca/, updated April 2, 2018), The Genetic Association Database (GAD, https://geneticassociationdb.nih.gov, updated August 18, 2014), Kyoto Encyclopedia of Genes and Genomes(KEGG, http://www.genome.jp/kegg, updated June 1, 2018), Online Mendelian Inheritance in Man (OMIM, http://www.omim.org, updated July 1, 2018), the Therapeutic Target Database(TTD, https://db.idrblab.org/ttd/, updated September 15, 2017).

#### 2.5.3 Protein–protein interaction analysis(PPI) and hub gene screening

The PPI of targets was generated by Bisogenet, a plug‐in of Cytoscape. Hub gene was screened using six parameters, including betweenness centrality (BC), closeness centrality (CC), degree centrality (DC), eigenvector centrality (EC), local average connectivity‐based method (LAC) and network centrality (NC).

#### 2.5.4 Pathway and biological functional enrichment analysis of Hub targets

Enrichment analysis of the Hub targets was performed by ClueGo, a plug‐in of Cytoscape (https://cytoscape.org), pV <0.05, Pathway = wikipathway. Visualisation was organized and imported using Cytoscape. Biological processes (BP) and pathways were visualized to show the relations between oridonin and gastric cancer targets.

### 2.6 Western blot assay^31^, ^35^

SGC‐7901 cells treated with oridonin were collected, and lysed with buffer (50 mM Tris‐Cl, pH 8.0, 120 mM NaCl, 50 mM NaF, 200 μM sodium vanadate, 0.5% NP‐40, 10 mM PMSF, 2 μg/mL aprotinin 0.2 μL, 10 μg/mL leupeptin 10 μL). The solution of the cell being lysed was centrifuged at 12000 × g at 4°C and the supernatant was collected. Protein concentration of the lysates were detected by Bradford assay. SDS‐PAGE was performed until the protein bands were completely separated, and then the proteins were transferred onto NC membranes. After blocking, the corresponding primary and secondary antibodies were added. The bands were measured by Gel Imaging System (GE, ImageQuant LAS500), and all the bands were repeated for three times.

### 2.7 Statistical analysis

Data are represented by mean ± standard deviation. The statistical analysis of data were calculated using Student's *t*‐test. *p* < 0.05 is believed as statistically significant.

## 3 RESULTS

### 3.1 Inhibition effect of oridonin on SGC‐7901 cells proliferation

Oridonin showed an inhibitory effect on SGC‐7901 cells and the proliferation inhibition rate increased gradually with the extension of drug treatment time (24, 48, 72 h) (Table 1, Figure 2B). The IC_50_ value was 6.55 × 10^1^ μM (Table 2). Cell morphology studies showed that cells in the control group showed up as irregular polygons with clear edges, large cell density, uniform arrangement and almost no suspended cells present (Figure 2C a). However, with the increase of the dosage of oridonin, the number of living cells (polygonal cells) gradually decreases and the number of dead cells (round cells) gradually increased (Figure 2C c,d,e). The morphology of the nuclei in the control was irregular oval, with moderate fluorescence intensity (Figure 2D a), while the cells nuclei treated with oridonin showed irregular shapes such as pyknosis, fragmentation, renal shape et al., and the fluorescence emitted by the cells is highlighted, which are characteristics of dying cells (Figure 2D c,d,e).

**TABLE 1:** Effect of oridonin on inhibition rate of the gastric cancer SGC‐7901 cell(x ± s, *n* = 6).

Group	Dosage (μΜ)	Inhibition rate (%)		
		24 h	48 h	72 h
Control	0	—	—	—
Oridonin	40	10.49 ± 1.83**	24.20 ± 5.13**	19.31 ± 1.57**
	50	12.29 ± 3.51**	28.68 ± 4.39**	28.44 ± 1.36**
	60	14.22 ± 3.66**	33.87 ± 4.86**	37.22 ± 3.33**
	70	15.25 ± 2.19**	40.69 ± 3.63**	48.71 ± 3.98**
	80	15.83 ± 2.50**	46.80 ± 7.81**	63.52 ± 6.10**
	90	21.94 ± 5.60**	63.24 ± 5.56**	81.80 ± 2.94**
	100	29.47 ± 3.59**	76.85 ± 2.94**	89.78 ± 0.94**
	110	40.03 ± 3.92**	84.19 ± 1.20**	91.26 ± 0.67**
	120	49.81 ± 1.43**	85.68 ± 0.54**	91.61 ± 0.31**

*Note*: compared with the control.

* *p* < 0.05

** *p* < 0.01.

**TABLE 2:** The IC_50_ of oridonin on the gastric cancer SGC‐7901 cell.

Groups	IC_50_ (μM)
Adr	1.88 × 10^−1^
Oridonin	6.55 × 10^1^

### 3.2 Potential targets of oridonin

The Pharmapper Database was adopted to identify the potential targets of oridonin, and the top 300 targets were selected as relevant. After processing, a total of 293 targets meeting the standards were obtained (Table S1).

### 3.3 Gastric cancer targets

Through five different databases, 180 targets relating to gastric cancer was identified (Table S3). A total of 18 targets were found by Drugbank, 56 targets were sought out from GAD, 103 targets were got from KEGG, 12 targets were mined from OMIM and 7 targets were fished using TTD.

### 3.4 Common targets fishing of oridonin in the treatment of gastric cancer and Hub target screening

By means of PPI, 7046 potential targets of oridonin (Figure3Aa, Table S2) and 5613 targets of gastric cancer related diseases (Figure 3Ab, Table S4) were obtained. Then a total of 4342 common targets of oridonin for gastric cancer were fished (Table S5). DC, BC, CC, EC, LAC, NC and other parameters were adopted to find 4342 common targets of oridonin for gastric cancer successively and finally 358 oridonin Hub targets for gastric cancer were obtained (Figure 3Ae, Table S6).

**FIGURE 4:**
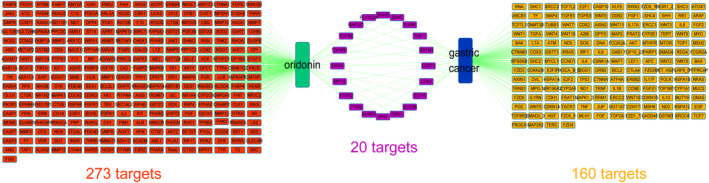
Oridonin‐target‐gastric Cancer networks. oridonin targets (red), gastric cancer targets (yellow), and overlapped targets (purple) accounted for 273, 160 and 20, respectively.

**FIGURE 5:**
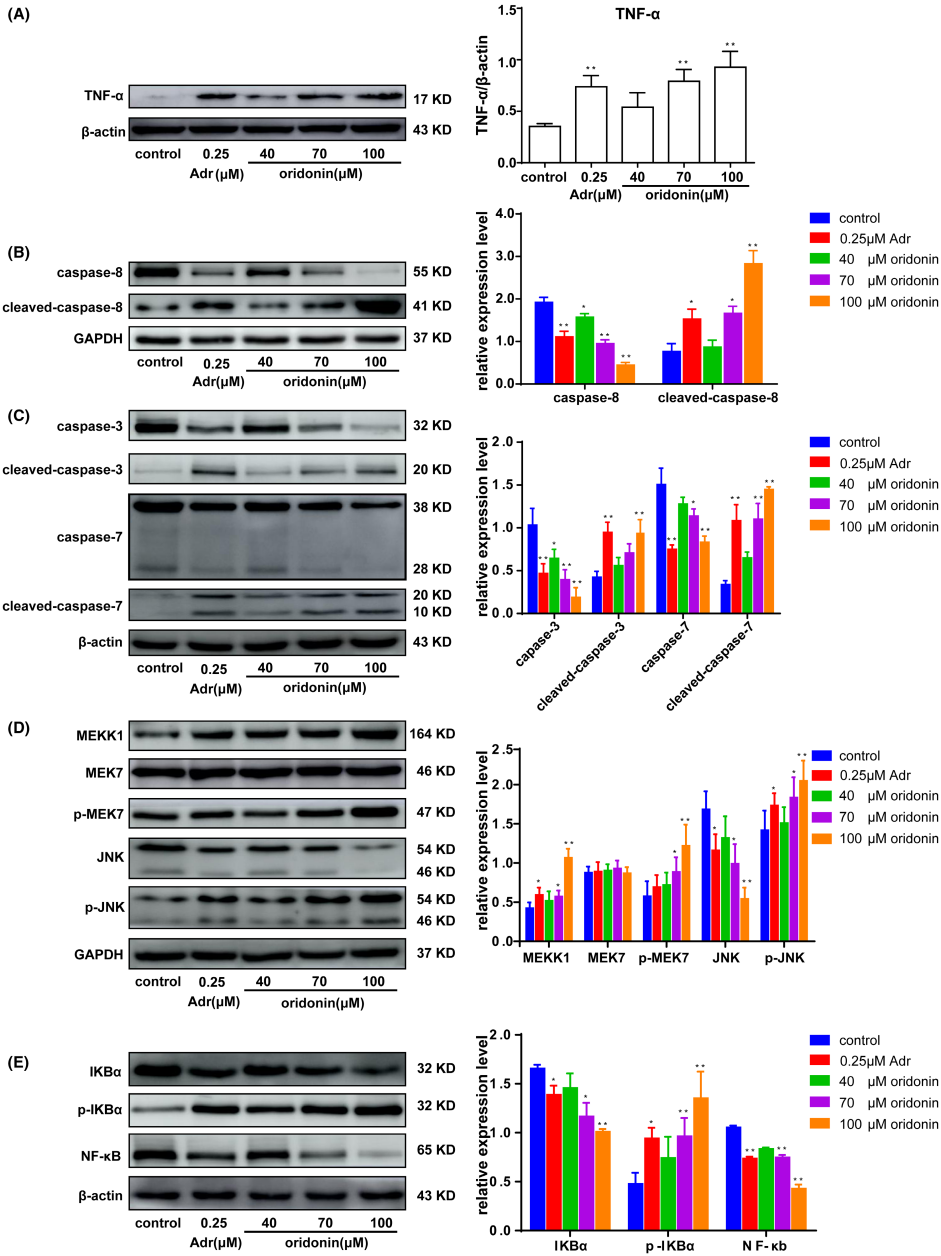
Effects of oridonin on expression of TNF‐α signalling pathway related proteins in human gastric cancer SGC‐7901 cells. (A)TNF‐α protein expression. (B) caspase‐8 protein expression. (C) caspase‐3 and caspase‐7 protein expression. (D) MEKK1, MEK7 and JNK protein expression; (E) NF‐κΒ protein expression.

**FIGURE 6:**
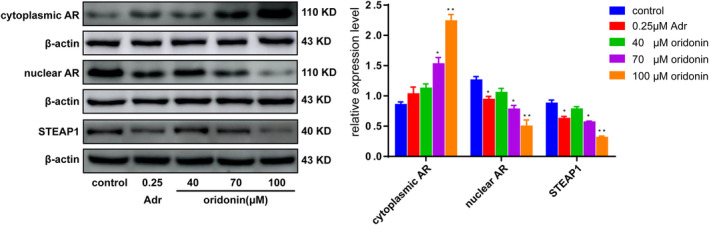
Effects of oridonin on expression of AR signalling pathway related proteins in human gastric cancer SGC‐7901 cells.

**FIGURE 7:**
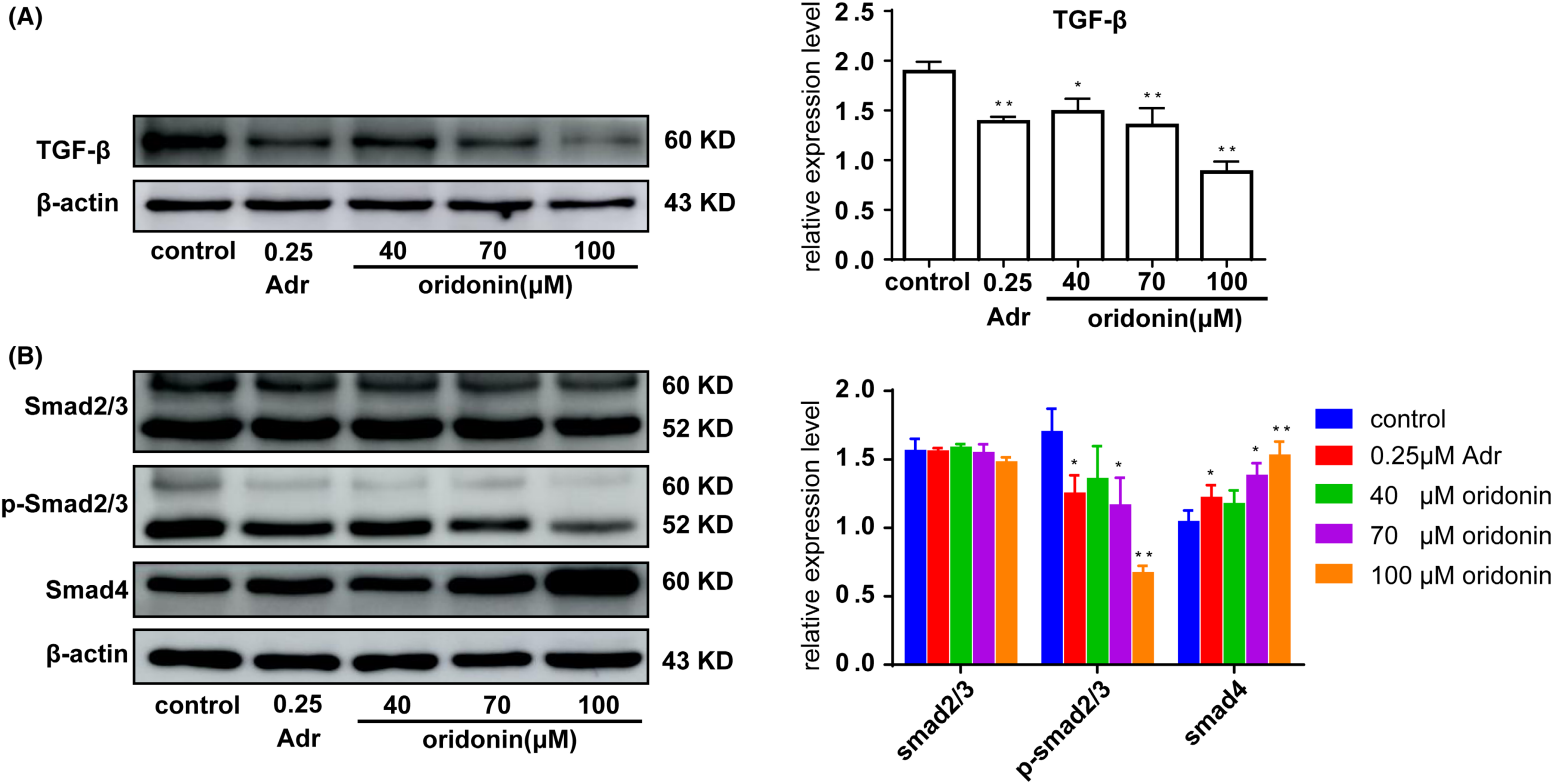
Effects of oridonin on expression of TGF‐β signalling pathway related proteins in human gastric cancer SGC‐7901 cells. (A) TGF‐β protein expression. (B) Smad2/3, p‐Smad2/3 and Smad4 protein expression.

**FIGURE 8:**
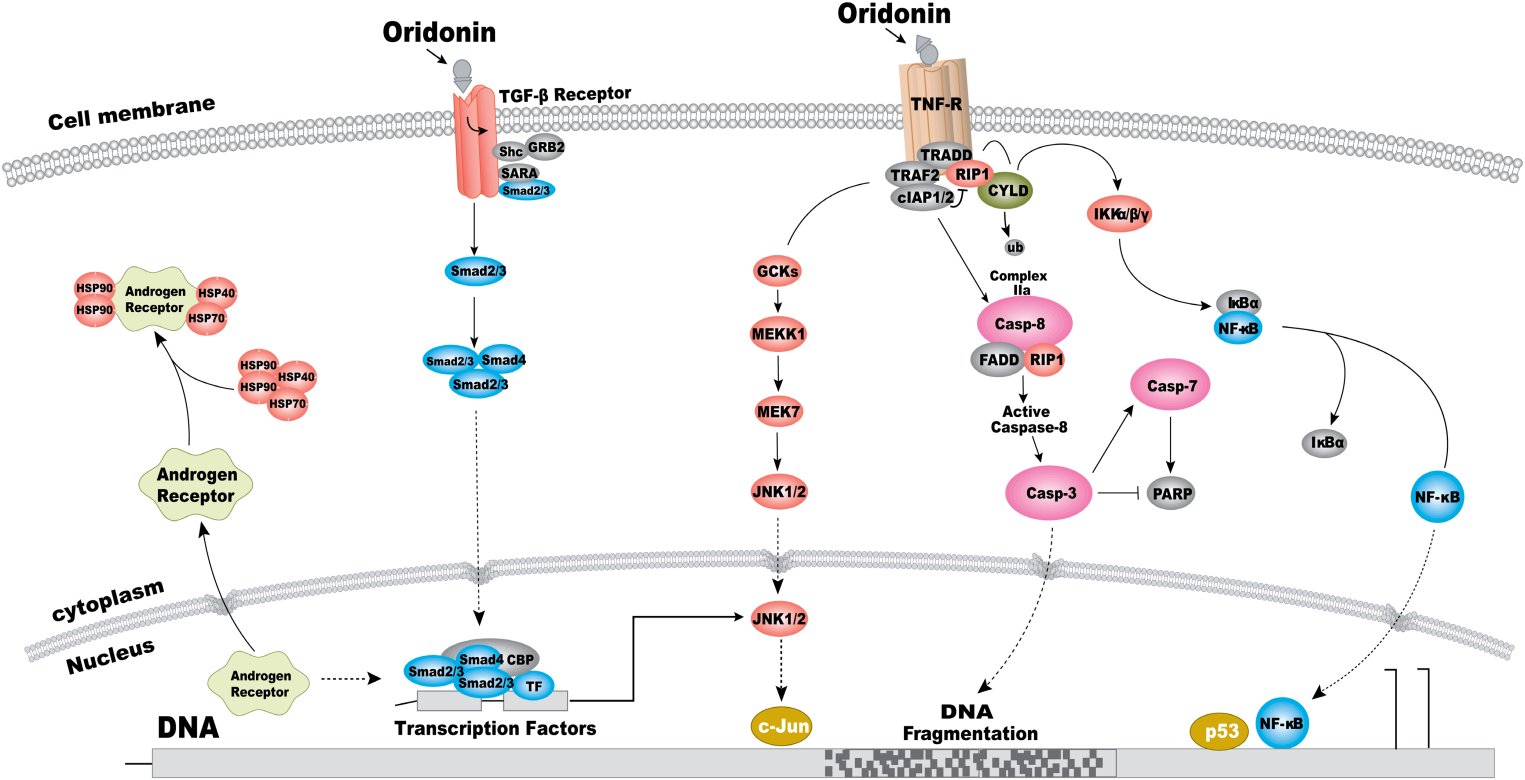
Potential signalling pathway of oridonin on gastric cancer constructed in the present research.

### 3.5 Oridonin‐Targets‐Gastric Cancer network construction

A network depicting Oridonin‐Targets‐Gastric Cancer was constructed to observe the anti‐gastric cancer mechanism of oridonin (Figure 4). The network consists of 453 nodes (273 oridonin targets, 160 gastric cancer‐related targets and 20 overlapping targets) and 473 edges. The 20 overlapped targets are the potential targets for the treatment of gastric cancer with oridonin, which suggested a possible mechanism for the treatment of on gastric cancer with oridonin.

### 3.6 Pathway enrichment analysis of the Hub targets

The 358 Hub targets of oridonin on gastric cancer were adopted to fish related pathways by wikipathway. 11 related pathways were enriched, among which the three pathways with the largest weight were TNF‐α signalling pathway, androgen receptor signalling pathway and TGF‐β signalling pathway (Figure 3B b). The results showed the anti‐gastric cancer effect of oridonin is potentially associated with the three signalling pathways.

### 3.7 Effects of oridonin on TNF‐α signalling pathway in SGC‐7901 cells

After oridonin intervention, the expression of TNF‐α protein increased with the increase of oridonin dosage (Figure 5A). The protein expressions of caspase‐8, caspase‐3 and caspase‐7 decreased and cleaved‐caspase‐8, cleaved‐caspase‐3, and cleaved‐caspase‐7 protein expression levels increased with an increasing dosage. These results suggest that oridonin can activate intracellular expression of cleaved‐caspase‐8, cleaved‐caspase‐3 and cleaved‐caspase‐7 proteins (Figure 5B,C).

The expression of MEK7 protein did not change significantly, while the expression of MEKK1, p‐MEK7 and p‐JNK protein gradually increased and the expression of JNK protein gradually decreased with the increase of oridonin dosage (Figure 5D).With the increase of oridonin dosage, the protein expression of IKBα and NF‐κB decreased gradually, while the protein expression of p‐IKB α increased gradually (Figure 5E).

### 3.8 Effects of oridonin on AR signalling pathway in SGC‐7901 cells

The protein expression of AR in cytoplasm was increased, while it was decreased in the nucleus, and the protein expression of STEAP1 in cells was also decreased, suggesting that oridonin could promote the flow of AR protein in the nucleus to the cytoplasm. It demonstrated the translocation of AR was induced by oridonin (Figure 6).

### 3.9 Effects of oridonin on the TGF‐β signalling pathway in SGC‐7901 cells

The expression levels of TGF‐β and p‐Smad 2/3 protein were decreased and the Smad4 protein were increased, but the Smad2/3 protein were not changed by oridonin, The results showed that oridonin could regulate down the TGF‐β expression, inhibiting the activation of Smad2/3 protein and activating Smad4 protein (Figure 7).

## 4 DISCUSSION

*Rabdosia rubescens* (Hemsl.) H.Hara has been used as a herb for many years in China. In Henan or Shanxi Provinces of China, local villagers often drink its decoction as a health promoting tea, which has a good therapeutic effect on chronic pharyngitis and tonsillitis. Villagers also treated cardia cancer by taking decoction of *Rabdosia rubescens* (Hemsl.) H.Hara for many years and had a good therapeutic effect. As one of the active components of *Rabdosia rubescens* (Hemsl.) H.Hara, oridonin has shown obvious anti‐cancer activity on various cancer cells, such as digestive system malignant tumours,^6^, ^26^ breast cancer^12^, ^13^ and prostate cancer,^20^ to name just a few. Angela M. Wong et al. compared the activity of the extract of Rabdosia rubescens (Hemsl.) H.Hara and oridonin on the anti‐tumour. The results showed that five times the pure oridonin more than it in the extract was required to obtain equivalent proliferation inhibition, which showed that oridonin is one of the anti‐cancer active compound in the Rabdosia rubescens (Hemsl.) H.Hara.^36^

At present, the relevant literature on the antitumor effects of oridonin are focused on the following aspects: inducing apoptosis by arresting the cell cycle,^37^ inducing DNA damage,^38^, ^39^ inducing autophagy,^15^ inhibiting tumour angiogenesis^3^ and inhibiting the migration of tumour cells.^18^, ^19^, ^40^ It is relatively single‐sided, and lacks comprehensive and multi‐angle research. The network pharmacology has broken the barriers in the study of traditional Chinese medicine, which makes it easier for us to apply the technology of experimental science to study traditional Chinese medicine.^34^ It provides a good idea for comprehensively elucidating the mechanism of drug action for wider applications.^41^

Oridonin can induce apoptosis of SGC‐7901 cells, inhibit cell proliferation and arrest cells in G_2_/M phase.^30^, ^31^ Other mechanisms however, still remain unclear. In this study, the results confirmed that oridonin can inhibit the proliferation of SGC‐7901 cells. Then, using the network pharmacology method, the proliferation of gastric cancer was inhibited by the oridonin through 11 signalling pathways, among which TNF‐α, AR and TGF‐β signalling pathways were the three most important signalling pathways. Finally, western blotting was adopted to determine the regulation of oridonin on the pathway axis composed of the three major signalling pathways and to explain the mechanism of oridonin in the treatment of gastric cancer.

A growth curve study found that oridonin could inhibit the proliferation of human gastric cancer SGC‐7901 cells. The IC_50_ value is 65.5 μM. The morphological study indicated that oridonin could damage the cell membrane and reduce the number of living cells. The observation of nuclear morphology proved that oridonin caused the typical nuclear morphology of cell death, such as nuclear fragmentation and dense staining.

In this study, through the study of network pharmacology, it was predicted that the proliferation inhibition effect of oridonin was related to the regulation of TNF‐α, AR and TGF‐β signalling pathways. Therefore, this finding was then experimentally verified in the following study.

TNF‐α is involved in many physiological and pathological processes, such as cellular proliferation or differentiation, regulation of immunologic, vascular invasion and destruction of tumour vasculature.^42^ TNF‐α binds to TNFR to activate caspase‐8, which activates downstream caspase‐3 and caspase‐7 protein through a cascade reaction, thereby inducing apoptosis; NF‐κB usually binds to inhibitory factor IκB in an inactive state in cells. Stimulation of NF‐κB results in the degradation of IκB and activation of NF‐κB. JNK is activated by the pro‐inflammatory factor TNF‐α. The activation program is that MAPKKK activates MAPKK, and MAPKK activates MAPK, then MAPK activates JNK signalling pathway.^43^ In this study, we found that oridonin up‐regulated TNF‐α protein expression and stimulated downstream caspase, JNK and NF‐κB signalling pathways. Activing caspase‐8, caspase‐3 and caspase‐7 can induce apoptosis. MAPK is an important signalling system that mediates cell biological responses. JNK is one of four MAPK signalling systems (ERK, JNK, P38, MAPK)^44^, ^45^, ^46^ in mammalian cells. MAPK signal transduction is completed through the MAPKKK‐MAPKK‐MAPK pathway, while MEKK1 is the MAPKKK within the cytoplasm. Through the MAPKKK‐MAPKK mode, MKK7 is activated^47^ and then the JNK pathway is activated.^48^ In this study, the results showed that oridonin increased the expression of MEKK 1, p‐MEK 7 and p‐JNK proteins in cells, suggesting that TNF‐α activated MEKK 1 and further activated MEK 7 under the action of oridonin. MEK 7 specifically activated JNK and promoted apoptosis; The expression of p‐IKBα was up‐regulated and NF‐κB was down‐regulated with the increase of oridonin dosage, suggesting that phosphorylation of IKBα was induced after TNF‐α stimulation. IKBα was an inhibitory factor of NF‐κB, so the activity of NF‐κB was inhibited, and the activity of promoting proliferation and inhibiting apoptosis was decreased. These findings showed that the inhibitory effect of oridonin on SGC‐7901 cells is related to TNF‐α signalling pathway, which is achieved by regulating caspase family proteins, NF‐κB signalling family proteins and promoting the protein expression of JNK signalling pathway.

AR is the androgen receptor, and AR signalling pathway is related to many hormone‐dependent diseases such as prostate cancer,^49^ breast cancer^50^, ^51^, ^52^ and ovarian cancer,^53^ as well as malignant tumours, for example gastric cancer,^54^, ^55^ lung cancer,^56^, ^57^ bladder cancer,^58^, ^59^ pancreatic cancer,^60^, ^61^ liver cancer^62^ and kidney cancer.^63^ The distribution of AR in cells is closely related to the survival of tumours. The combination of androgen and AR occurs in the nucleus, so the distribution and metastasis of AR in cytoplasm and nucleus are closely related to the proliferation of tumours. STEAP1 protein downstream of AR signalling pathway exists at the junction of cell membranes and acts as a ‘communicator’ transmitting information between cells.^64^, ^65^ The results showed that oridonin up‐regulated cytoplasmic AR protein expression, down‐regulated nuclear AR protein and downstream STEAP 1 protein. In other words, oridonin can regulate the distribution of AR between the cytoplasm and nucleus, inhibit the transport of AR from cytoplasm to nucleus, or promote the flow of AR from nucleus to cytoplasm and then affect the binding amount of androgen to AR in nucleus, thus affecting the proliferation of SGC‐7901 cells.

TGF‐β is a growth transformation factor, not only involved in tumour proliferation,^66^ invasion,^67^, ^68^ migration,^69^ adhesion,^70^, ^71^ induce cell cycle arrest^72^, ^73^ and apoptosis,^74^, ^75^ but also through up‐regulation of cell growth inhibitory factors and inflammatory factors, so as to achieve the purpose of inhibiting tumour proliferation. Smads are intracellular signal transduction molecules, which can transfer TGF‐β signal from the cell membrane to the nucleus. When its function is abnormal, TGF‐β signal transduction will also be affected. TGF‐β binds to its receptor and phosphorylates. The activated TβRI can specifically recognize downstream Smad2 and Smad3, bind to and activate them, and the activated Smad2 and Smad3 dissociate from TβRI and then form heterologous complex with tumour suppressor gene Smad4. It is then transferred into the nucleus to affect the expression of target genes.^76^, ^77^ Oridonin could down‐regulate TGF‐β and p‐smad2/3 protein expression and up‐regulate Smad4 protein expression, thereby reducing downstream Smad2/3 activity, activating the Smad4 protein and inhibiting the proliferation of cancer cells.^78^ Hence, SGC‐7901 cell proliferation inhibited is related to its regulation of the TGF‐β signalling pathway.

Tumour‐related signalling pathways are not only independent but also indivisible. They interweave, interact and influence each other to jointly affect the tumours and participate in the regulation of tumour cell proliferation, differentiation, invasion, metastasis, angiogenesis, immune response as well as other mechanisms. JNK is not only activated by TNF‐α, but also closely related to the caspase family, NF‐κB pathway and TGF‐β pathway. JNK mediates the exogenous death receptor pathway initiated by caspase‐8, and its active substance promotes the release of mitochondrial pro‐apoptotic protein jBID, which promotes the release of caspase‐8 inhibitors on the TNFR1 complex. Thus, the inhibition of caspase‐8 was decreased, and apoptosis was induced.^79^ JNK is also activated by TGF‐β in many cancer cell lines, and the TGF‐β/Smad is upstream of JNK. MEKK1, a member of the MAPKKK family, directly binds to and activates the NF‐κB inhibitory kinase signals IKKα and IKKβ, leading to phosphorylation. MEKK1 is also an upstream kinase of IκB, selectively phosphorylating the Ser32 and Ser36 sites of IκB, which together activate the downstream NF‐κB to accelerate proliferation and reduce apoptosis.^80^, ^81^ That is, TNF‐α pathway, androgen receptor pathway and TGF‐β pathway are independent but related to each other and the three pathways constitute a TNF‐α/AR/TGF‐β signalling pathway axis.

## 5 CONCLUSIONS

In conclusion, in this study, the signal pathway of oridonin inhibiting gastric cancer proliferation was predicted by network pharmacology. Experimental studies confirmed that the proliferation inhibition of oridonin on SGC‐7901 cell was related to TNF‐α/AR/TGF‐β signalling pathway axis, which confirmed the prediction of network pharmacology, and elucidated the mechanism of oridonin on gastric cancer (Figure 8).

## AUTHOR CONTRIBUTIONS

**Shiyong Gao:** Conceptualization (lead); funding acquisition (equal); investigation (equal); software (equal); writing – original draft (equal). **Huixin Tan:** Data curation (equal); project administration (equal); writing – original draft (equal); writing – review and editing (equal). **Dan Li:** Investigation (equal); methodology (equal); writing – review and editing (equal).

## FUNDING INFORMATION

Natural Science Foundation in Heilongjiang Province, Grant/Award Number: LH2021H002; 2021 Harbin University of Commerce Teacher “Innovation” Project, Grant/Award Number: LH2021H002; Outstanding Young Talents Project of the Central Government's Fund for Local Universities' Reform and Development, Grant/Award Number: 2020YQ12; Science and Technology Project of Heilongjiang Provincial Health and Family Planning Commission, Grant/Award Number: 2017–132; General Project of Education Department of Heilongjiang Province, Grant/Award Number: 12541571.

## CONFLICT OF INTEREST STATEMENT

The authors declare that they have no conflict of interest.

## Supporting information


Table S1.

Table S2.

Table S3.

Table S4.

Table S5.

Table S6.

## Data Availability

The data that supports the findings of this study are available in the supplementary material of this article
